# The clinical course of alcoholic cirrhosis: effects of hepatic metabolic capacity, alcohol consumption, and hyponatremia – a historical cohort study

**DOI:** 10.1186/1756-0500-5-509

**Published:** 2012-09-18

**Authors:** Peter Jepsen, Peter Ott, Per Kragh Andersen, Hendrik Vilstrup

**Affiliations:** 1 Department of Medicine V (Hepatology and Gastroenterology), Aarhus University Hospital, Aarhus, Denmark; 2 Department of Clinical Epidemiology, Aarhus University Hospital, Aarhus, Denmark; 3 Department of Biostatistics, Institute of Public Health, University of Copenhagen, Copenhagen, Denmark

**Keywords:** Alcoholic liver disease, Hepatic encephalopathy, Ascites, Variceal bleeding, Prognosis

## Abstract

**Background:**

The cirrhosis complications hepatic encephalopathy, ascites, and variceal bleeding increase mortality but develop in random sequence. Therefore prognoses based on the presence or absence of these clinical complications are inherently inaccurate, and other determinants of the clinical course should be identified. Here we present our study of patho-etiological factors that may be causally involved in the development of specific complications to alcoholic cirrhosis; it was based on a model of cirrhosis pathophysiology encompassing hepatic metabolic capacity, continued alcohol consumption, and circulatory dysfunction.

**Methods:**

We followed a Danish community-based cohort of 466 patients with alcoholic cirrhosis. Stratified Cox regression was used to examine the effects of GEC (a measure of hepatic metabolic capacity), alcohol consumption, and plasma sodium concentration (a measure of circulatory dysfunction) on the hazard rates of first-time hepatic encephalopathy, first-time ascites, first-time variceal bleeding, and mortality. We adjusted for confounding by comorbidity, gender, and age. Data on risk factors and confounders were updated during follow-up.

**Results:**

A low GEC increased the risk of first-time hepatic encephalopathy (hazard ratio [HR] 1.21 per 0.1 mmol/min GEC loss, 95% CI 1.11-1.31), but was unassociated with other adverse events. Alcohol consumption increased the risk of first-time ascites (HR 3.18, 95% CI 1.19-8.47), first-time variceal bleeding (HR 2.78, 95% CI 1.59-4.87), and mortality (HR 2.45, 95% CI 1.63-3.66), but not the risk of first-time hepatic encephalopathy. Hyponatremia increased the risk of all adverse events.

**Conclusions:**

Reduced hepatic metabolic capacity, alcohol consumption, and hyponatremia were causally involved in the development of specific complications to alcoholic cirrhosis.

## Background

We recently described the development of hepatic encephalopathy, ascites, and variceal bleeding in Danish patients with alcoholic cirrhosis [1]. The appearance of these complications increased mortality, but they developed in random sequence. Therefore mortality predictions based solely on their presence or absence are inherently inaccurate, and other determinants of the clinical course should be identified.

Cirrhosis patients have reduced hepatic metabolic capacity, and the residual capacity can be measured with the galactose elimination capacity (GEC) test [2], the result of which is a strong predictor of mortality [3]. Some patients with alcoholic cirrhosis continue drinking alcohol, and such exposure to the etiology of their disease increases mortality [4]. Finally, cirrhosis is accompanied by renal dysfunction and water dyshomeostasis causing hyponatremia which is associated with increased mortality [5]. We examined whether those three patho-etiological factors—reduced hepatic metabolic capacity, continued  alcohol  consumption,  and circulatory dysfunction—are causally involved in the development of specific complications to alcoholic cirrhosis, and not merely predictors of an elevated mortality. Our study was based on a model of cirrhosis pathophysiology restricted to the three patho-etiological factors and relevant confounders, and we aimed to establish causal links between the pathophysiology and the clinical course of alcoholic cirrhosis.

## Methods

We followed a community-based cohort of 466 Danish citizens who resided in our hospital’s catchment area and were diagnosed with alcoholic cirrhosis between 1 January 1993 and 31 August 2005 and had not previously been examined for suspected cirrhosis. Thirty patients (6%) were diagnosed on the basis of liver biopsy findings, the remainder on the basis of clinical, biochemical, imaging, and hemodynamic findings. Six patients received a liver transplant [1]. According to Danish law, registry-based studies such as this require neither ethical approval nor patient consent.

### Galactose elimination capacity, alcohol consumption, and plasma sodium concentration

The GEC is a physiological measure of hepatic metabolic capacity and is part of our clinical workup of patients with cirrhosis [2,3]. We obtained the results of all GEC tests conducted during the follow-up period from our department’s laboratory database. Data on patients’ alcohol consumption were obtained from the medical charts. We recorded patients’ current alcohol drinking status (abstinent or drinking) as reported at each hospital contact. Abstinence was defined as complete abstinence or consumption of small amounts of alcohol on rare occasions. Drinking was defined as non-abstinence. Plasma sodium concentrations were measured frequently in both in- and outpatients, and we extracted all measurements from the database of the department of clinical biochemistry.

### Confounders: comorbidity, gender, and age

Comorbidity, male gender, and older age increase mortality among cirrhosis patients [6,7], and these factors are also associated with hepatic metabolic capacity [3]. They were therefore considered potential confounders of the associations of interest in this study. Information on patients’ comorbidities were extracted from the Danish National Patient Registry and scored with the Charlson Comorbidity Index (CCI), which assigns a weight to chronic diseases according to their impact on mortality [6]. Comorbidities diagnosed during follow-up were added to the CCI from their date of diagnosis.

### Outcome events

We examined four outcome events: first-time hepatic encephalopathy, first-time ascites, first-time variceal bleeding, and death. Data were obtained from the patients’ medical charts and from the Danish Civil Registration System which is continuously updated with dates of birth, death, and migration for the Danish population [8]. Hepatic encephalopathy was defined as overt hepatic encephalopathy, and ascites was defined as clinically detectable ascites, i.e., ascites detected only on ultrasound examination was excluded. Variceal bleeding was defined as clinically unequivocal bleeding from esophageal or gastric varices with hematemesis in combination with a heart rate >100 beats per minute and systolic blood pressure <100 mmHg or a need for blood transfusion.

### Statistical analysis

We conducted four separate analyses to examine the risk factors for the four events of interest. In each analysis the patients were followed from the diagnosis of cirrhosis until they experienced the event. Event-free patients were censored on 31 August 2006, and—in the three analyses with a complication as the event of interest—also at death. We used stratified Cox proportional hazards regression with GEC, alcohol consumption, plasma sodium concentration, CCI, and age as time-dependent variables [9], gender as a time-fixed variable, and the patients’ current complications as time-dependent strata. The stratification implied the assumption that, for example, patients with hepatic encephalopathy and patients with ascites had different mortality rates (and patients with variceal bleeding or no complications had yet other mortality rates), but a 0.1 mmol/min GEC loss had the same multiplicative effect on everybody’s mortality rate. Not all patients had a GEC test, so the analyses were conducted with and without GEC included in the regression model. In the analyses including GEC we included only patients who had a GEC test within 30 days after cirrhosis diagnosis, and patients were followed from the date of the GEC test. Effect estimates whose 95% confidence interval excluded the null value were considered statistically significant. The assumption of proportional hazards was tested with Schoenfeld residuals against follow-up time and was not violated.

## Results

The study comprised 466 newly diagnosed alcoholic cirrhosis patients: 114 (24%) without complications, 19 (4.1%) with hepatic encephalopathy alone, 254 (55%) with ascites alone, 29 (6.2%) with variceal bleeding alone, 23 (4.9%) with hepatic encephalopathy and ascites, 2 (0.4%) with hepatic encephalopathy and variceal bleeding, 20 (4.3%) with ascites and variceal bleeding, and 5 (1.1%) with all three complications. Hence, 114 + 254 + 29 + 20 = 417 patients did not have hepatic encephalopathy and were consequently at risk of first-time hepatic encephalopathy; similarly, 164 patients were at risk of first-time ascites, and 410 patients were at risk of first-time variceal bleeding (Table 1).

**Table 1:** Patient characteristics at diagnosis of alcoholic cirrhosis

	**At risk of first-time hepatic encephalopathy**	**At risk of first-time ascites**	**At risk of first-time variceal bleeding**	**All patients**
Number of patients	417	164	410	466
Total follow-up time	1481 years	553 years	1420 years	1611 years
*Complications at cirrhosis diagnosis*^†^				
None	114 (27%)	114 (70%)	114 (28%)	114 (24%)
Hepatic encephalopathy	-	21 (13%)	42 (10%)	49 (11%)
Ascites	274 (66%)	-	277 (68%)	302 (65%)
Variceal bleeding	49 (12%)	31 (19%)	-	56 (12%)
GEC, median (25^th^ – 75^th^ percentile)	1.59 (1.36-1.88)	1.75 (1.48-2.06)	1.55 (1.34-1.79)	1.56 (1.35-1.86)
Alcohol consumers (%)	327 (78%)	124 (76%)	313 (76%)	366 (79%)
Plasma sodium, median (25^th^ – 75^th^ percentile)	136 (132–139)	138 (135–140)	136 (132–139)	136 (132–139)
Charlson Comorbidity Index > 0	157 (38%)	76 (46%)	153 (37%)	177 (38%)
Age, median (25^th^ – 75^th^ percentile)	51 (45–60)	53 (47–61)	53 (46–61)	53 (47–61)
Men (%)	295 (71%)	123 (75%)	284 (69%)	329 (71%)
*Events during follow-up*				
First-time hepatic encephalopathy	120 (29%)^#^	19 (12%)	75 (18%)	120 (26%)
First-time ascites	45 (11%)	55 (34%)^#^	41 (10%)	55 (12%)
First-time variceal bleeding	78 (19%)	18 (11%)	91 (22%)^#^	91 (20%)
Death	150 (36%)	57 (35%)	192 (47%)	299 (64%)

^†^ Patients may have more than one complication.

^#^ Follow-up stopped when this event occurred.

A low GEC increased the risk of first-time hepatic encephalopathy (adjusted hazard ratio = 1.21 per 0.1 mmol/min GEC loss, 95% CI = 1.11-1.31), but did not affect the risk of first-time ascites or first-time variceal bleeding or increase mortality. Alcohol consumption had opposite effects, i.e., it was not a risk factor for first-time hepatic encephalopathy but increased the risks of first-time ascites, first-time variceal bleeding, and mortality. Hyponatremia was a risk factor for all three complications and also increased mortality. Associations were the same in the full cohort and in the subcohort with a GEC test (Table 2).

**Table 2:** Effects of galactose elimination capacity, alcohol consumption, and plasma sodium concentration on the clinical course of alcoholic cirrhosis

	**Hepatic encephalopathy**		**Ascites**		**Variceal bleeding**		**Death**	
	**All patients at risk**	**Patients at risk + with GEC test**	**All patients at risk**	**Patients at risk + with GEC test**	**All patients at risk**	**Patients at risk + with GEC test**	**All patients at risk**	**Patients at risk + with GEC test**
	**N = 417**	**N = 237**	**N = 164**	**N = 56**	**N = 410**	**N = 231**	**N = 466**	**N = 266**
GEC, per 0.1 mmol/min loss		**1.21 (1.11-1.31)**		1.04 (0.84-1.30)		1.01 (0.95-1.08)		1.04 (0.98-1.10)
Alcohol consumption	0.93 (0.51-1.70)	0.82 (0.45-1.50)	**1.95 (1.16-3.26)**	**3.18 (1.19-8.47)**	**2.01 (1.28-3.14)**	**2.78 (1.59-4.87)**	**2.20 (1.68-2.88)**	**2.45 (1.63-3.66)**
Plasma sodium, per mmol/L loss	**1.14 (1.10-1.19)**	**1.14 (1.09-1.18)**	**1.12 (1.05-1.20)**	**1.16 (1.06-1.28)**	**1.08 (1.05-1.11)**	**1.07 (1.04-1.10)**	**1.13 (1.11-1.15)**	**1.14 (1.11-1.18)**

Effects are expressed as hazard ratios controlled for confounding by the two other patho-etiological measures, gender, age, and Charlson comorbidity index. Statistically significant associations are highlighted with bold font.

## Discussion

Our community-based study of 466 patients with newly diagnosed alcoholic cirrhosis supported our hypothesis that patho-etiological factors are causally involved in the development of specific complications. In particular, reduced hepatic metabolic capacity was specifically associated with the risk of developing first-time hepatic encephalopathy. Alcohol consumption increased the risk of all adverse events except the development of first-time hepatic encephalopathy. Hyponatremia, by increasing the risk of all complications as well as mortality, was a non-specific marker of a poor prognosis.

The major strength of our study is that it was designed to evaluate hypotheses about the *causal* effects of reduced hepatic metabolic capacity, continued alcohol consumption, and hyponatremia on the development of complications—not merely to examine whether they *predict* their development. A clear distinction between causes and predictors is necessary in order to advance our understanding of cirrhosis pathophysiology: A cause is always a predictor, but a predictor can be either a cause or a correlate of a cause, an ‘innocent bystander’ [10]. A composite score such as the MELD (Model for Endstage Liver Disease) score is a likely predictor of cirrhosis progression, but the causal effect(s) of a high MELD score are unclear because it may represent a loss of kidney function (high creatinine), a loss of hepatic synthetic capacity (high INR), and/or a loss of hepatic conjugative capacity (high bilirubin). Therefore we did not include the MELD score in our analyses. Nor did we include its components or other standard liver biochemistry tests because they correlate with the GEC but are not truly tests of hepatic metabolic capacity [11].

Given this background, our findings are consistent with the definition that loss of hepatic metabolic capacity is a prerequisite for developing hepatic encephalopathy [12], but we add that loss of hepatic metabolic capacity does not cause death without hepatic encephalopathy. This novel finding expands upon our previous finding that the GEC is a predictor of the mortality of cirrhosis patients [3]: It is now clear that loss of hepatic metabolic capacity causes hepatic encephalopathy and then death [1]. Our previous study suggested that a GEC above 1.75 mmol/min was associated with a relatively favorable prognosis, whereas mortality increased linearly with GEC when GEC fell below this limit [3]. We speculate that the same limit applies to the risk of hepatic encephalopathy, but our current study did not have sufficient statistical power to clarify this.

Alcohol consumption can cause ascites formation and variceal bleeding, according to our findings. The likely explanation is that alcohol intake induces a prompt increase in portal pressure [13-15]. However, the association might also be partly due to alcohol consumers’ noncompliance with diuretics and beta-blocker treatments, i.e. non-causal. The association between alcohol consumption and mortality was expected because alcoholism is a risk factor for death among infected cirrhosis patients and for several cancers [16,17]. It is even possible that we underestimated the adverse effects of alcohol because we may have missed alcohol relapse in some patients who died at home. The lack of an association between alcohol consumption and hepatic encephalopathy is consistent with findings from a randomized clinical trial comparing porto-systemic shunt placements. In that study, drinking spells during follow-up were more strongly associated with variceal bleeding and mortality than with hepatic encephalopathy [18].

The cirrhosis patients with hyponatremia had higher risk of *all* adverse events, so these patients’ clinical course remained as unpredictable as that of patients without hyponatremia. Our findings are consistent with observations that hyponatremia causes hepatic encephalopathy [10,19], but we might have learned more if we had measured urinary sodium excretion which decreases before dilutional hyponatremia develops [20]. Unfortunately, it was not measured in a meaningful proportion of our patients, and it remains possible that hyponatremia is an innocent bystander rather than an agent in the causal pathways to the development of cirrhosis complications.

Our data had high validity. We believe that the medical charts from one of Denmark’s two specialized departments of hepatology gave reliable records of all first-time episodes of major cirrhosis complications. The chart data were extracted previously for a different research purpose [1], so there was no bias from a conscious or unconscious wish to find certain associations in the data. The absence of data on portal pressure and medication use is a limitation of the study because both factors modify the risk of complications and represent distinct pathophysiological mechanisms.

## Conclusion

In conclusion, reduced hepatic metabolic capacity, continued alcohol consumption, and circulatory dysfunction had distinct adverse effects on the risks of first-time hepatic encephalopathy, first-time ascites, first-time variceal bleeding, and death. Our findings unite, expand, and strengthen the findings from several previous studies on the clinical course of cirrhosis and improve our understanding of the disease.

## Competing interests

The authors declare that they have no competing interests.

## Authors’ contributions

PJ conceived the study, and PJ and PKA designed it. PJ conducted the analyses, and all authors contributed to their interpretation. PJ drafted the manuscript, and all authors revised it for important intellectual content. All authors have approved the final manuscript.
